# Integrin αvβ3 in the Mediating Effects of Dihydrotestosterone and Resveratrol on Breast Cancer Cell Proliferation

**DOI:** 10.3390/ijms21082906

**Published:** 2020-04-21

**Authors:** Yih Ho, Zi-Lin Li, Ya-Jung Shih, Yi-Ru Chen, Kuan Wang, Jacqueline Whang-Peng, Hung-Yun Lin, Paul J. Davis

**Affiliations:** 1 School of Pharmacy, Taipei Medical University, Taipei 110, Taiwan; yiho@tmu.edu.tw; 2 Graduate Institute of Nanomedicine and Medical Engineering, College of Medical Engineering, Taipei Medical University, Taipei 110, Taiwan; lizilin0919@gmail.com (Z.-L.L.); shihyj@tmu.edu.tw (Y.-J.S.); aquarlus9132@gmail.com (Y.-R.C.); wangk007@gmail.com (K.W.); 3 Graduate Institute of Cancer Biology and Drug Discovery, Taipei Medical University, Taipei 110, Taiwan; jqwpeng@nhri.org.tw; 4 The PhD Program for Cancer Molecular Biology and Drug Discovery, College of Medical Science and Technology, Taipei Medical University, Taipei 110, Taiwan; 5 TMU Research Center of Cancer Translational Medicine, Taipei Medical University, Taipei 110, Taiwan; 6 Cancer Center, Wan Fang Hospital, Taipei Medical University, Taipei 116, Taiwan; 7 Traditional Herbal Medicine Research Center, Taipei Medical University Hospital, Taipei Medical University, Taipei 110, Taiwan; 8 Pharmaceutical Research Institute, Albany College of Pharmacy and Health Sciences, Rensselaer, NY 12208, USA; pdavis.ordwayst@gmail.com; 9 Department of Medicine, Albany Medical College, Albany, NY 12208, USA

**Keywords:** integrin αvβ3, dihydrotestosterone, resveratrol, breast cancer

## Abstract

Hormones and their receptors play an important role in the development and progression of breast cancer. Hormones regulate the proliferation of breast cancer cells through binding between estrogen or progestins and steroid receptors that may reside in the cytoplasm or be transcriptionally activated as steroid–protein nuclear receptor complexes. However, receptors for nonpeptide hormones also exist in the plasma membrane. Via those receptors, hormones are able to stimulate breast cancer cell proliferation when activated. Integrins are heterodimeric structural proteins of the plasma membrane. Their primary functions are to interact with extracellular matrix proteins and growth factors. Recently, integrin αvβ3 has been identified as a receptor for nonpeptide hormones, such as thyroid hormone and dihydrotestosterone (DHT). DHT promotes the proliferation of human breast cancer cells through binding to integrin αvβ3. A receptor for resveratrol, a polyphenol stilbene, also exists on this integrin in breast cancer cells, mediating the anti-proliferative, pro-apoptotic action of the compound in these cells. Unrelated activities of DHT and resveratrol that originate at integrin depend upon downstream stimulation of mitogen-activated protein kinase (MAPK, ERK1/2) activity, suggesting the existence of distinct, function-specific pools of ERK1/2 within the cell. This review will discuss the features of these receptors in breast cancer cells, in turn suggesting clinical applications that are based on the interactions of resveratrol/DHT with integrin αvβ3 and other androgen receptors.

## 1. Introduction

The theory that hormone binding to the cell surface can contribute to breast carcinogenesis was developed in large part from the discovery of plasma membrane estrogen receptors [1]. Integrins are heterodimeric structural proteins of the plasma membrane. Their primary roles are to promote cell–cell adhesion and the interactions of cells with extracellular matrix proteins. Integrin αvβ3 is expressed by cancer cells, as well as rapidly dividing endothelial and vascular smooth muscle cells. Recently, distinct receptors on the plasma membrane integrin αvβ3 of breast cancer (BC) cells have been described for thyroid hormone, resveratrol, and dihydrotestosterone (DHT). Integrins have been generally seen to bear receptors or binding sites only for relatively large molecules—extracellular matrix proteins and growth factors [2,3,4]—and thus it was surprising to find apparently biologically relevant binding sites for small molecules on integrins. Functions of these membrane receptors include modulating cancer cell proliferation and, in the case of the thyroid hormone, tumor-relevant angiogenesis.

Estrogen receptor (ER)–progesterone receptor (PR) status in breast carcinomas has three molecular subtypes: human epidermal growth factor receptor 2 (HER2) over-expression, ER and PR expression and absence of ER, and PR and HER2 expression (triple negative). These receptors may be considered targets for management of breast cancer proliferation. Recently, Giovannelli et al. also reported the role of sex steroid receptors (SSRs) in BC stem cells (BCSCs); although the growth and metastatic regulation of BCSCs is still unclear, SSRs expressed in BCSCs are considered as a marker of stemness and are linked to BC proliferation, as well as metastatic and malignant properties [5]. Overexpression of SSRs in BC usually suggests that their role is in the tumor microenvironment and the levels of circulating sex hormones; however, the SSRs’ function in BC is still conflicting [6]. In this review, we will describe the features of these receptors in breast cancers and propose clinical applications based on the interactions of resveratrol/DHT and integrin αvβ3, as well as other androgen receptors. The effects of steroid hormones and thyroid hormone and their receptors on cancer growth are summarized in Table 1.

## 2. Androgen and Other Non-Peptide Hormones Act via Different Receptors to Induce Proliferation of Human Breast Cancer Cells

In the traditional concept of steroid action, ERs and androgen receptors (ARs) exist in the cytosol and translocate into the nucleus after complexing with their respective ligands. The androgen–AR complex binds to the promoter of androgen-responsive genes. However, there is evidence for the existence of more than one cellular androgen binding site. The relative roles of ER and AR in breast cancer proliferation are controversial. For example, dehydroepiandrosterone sulfate (DHEAS) causes breast cancer cell proliferation via ERs, but can also inhibit proliferation through ARs [14,15].

The AR can be an important contributor to breast cancer cell proliferation, as Yeh et al. have shown [16]. Up to 85% of breast cancers express nuclear ARs [16]. In addition, 25%–82% of metastatic breast tumors that are ER- and PR-negative still express a significant number of ARs [16]. Most ER-positive breast tumors express ARs [15,17,18], and AR expression in ER-positive cases is associated with smaller tumor size, lower Nottingham grade, and less frequent tumor cell necrosis [19]. AR is also expressed in ER-negative/progesterone receptor (PR)-negative/HER2+ tumors, and in a subset of triple-negative apocrine tumors [19]. Approximately one in five breast cancer 1 (BRCA1) gene-expressing cancers also express Ars, but are negative for ERs and PRs [20]. However, our studies indicate that the detectable ARs in ER-negative MDA-MB-231 cells may not be functional for DHT, since there is no proliferation by DHT in the presence or absence of AR siRNA [7].

Steroid hormones and their receptors are implicated in the pathogenesis of breast cancers [21]. Studies further indicate that the interaction of the AR–ligand and co-activator plays an important role in gene expression. The AR co-activator, p44/Mep50, a subunit of the methylosome complex, enhances AR-mediated transcription activity in a ligand-dependent manner [22,23]. While it may act as a nuclear co-activator in breast cancer cells, p44 is also present in substantial quantities in the cytoplasm of terminal ductal lobular units [22,23]. When overexpressed by MCF7 breast cancer cells, p44 has been shown to enhance proliferation and invasiveness [22].

The other nuclear receptor co-activator related to invasion is actin-binding protein, actinin α 4 (ACTN4), which has been shown to promote the proliferation of MCF-7 breast cancer cells [24]. Knockdown of ACTN4 reduces transcription of ERα target genes and modulates MCF-7 cell proliferation in the absence of estrogen [24]. In late-stage metastatic breast cancers, the ACTN4 levels decrease in the nucleus, as is observed in high-grade cancerous prostate samples, suggesting that ACTN4 is possibly deregulated in advanced stage cancers [25]. ACTN4 and protein kinase C δ (PKC δ) display both co-activator and co-repressor activity in the process of AR-mediated transcription, whereas clathrin heavy chains exhibit co-activator activity during AR-mediated transcription [25].

## 3. Membrane Androgen Receptors

Breast and prostate cancer cells express membrane androgen receptors (mARs) subject to control by specific ligands. A panel of essential functions of these cells—proliferation, cell motility, and susceptibility to apoptosis—is regulated by such ligands [26,27] mAR-linked actions can readily be distinguished from those initiated or mediated by classical intracellular androgen receptors (iARs) by certain anti-androgens [27]. Testosterone analogues that are excluded from the cell interior, e.g., testosterone–bovine serum albumin (BSA), may express significant androgen-related biologic actions in breast cancer cells that contain iARs [28,29]. Stimulation of colon mAR by the testosterone–BSA conjugate induces rapid cytoskeleton reorganization and apoptotic responses, even in the presence of anti-androgens [26]. Apoptosis that is testosterone-induced is related to p38 MAPK and phosphatidylinositol-3-kinase (PI-3K)/Akt/NF-κB or Rho/actin pathways. The JNK/c-JUN signaling pathway appears to mediate certain iAR-initiated events [29]. The non-permeable testosterone–BSA conjugate binds mARs to stimulate early actin reorganization, which is regulated by early phosphorylation of focal adhesion kinase (FAK) and subsequent PI-3K and Rac1 activation.

Acting on breast fibroblasts in vitro, testosterone has been shown by Quinn and co-workers to enhance estrogen-responsive pS2 gene transcription and the generation of estradiol via aromatase activity in the medium [30]. Addition of an aromatase inhibitor blocked production of fibroblast-source estrogen and modestly increased cell pS2 transcription [30]. It is not yet clear what the clinical significance may be of the AR on the cell surface of breast cancer cells. However, when the action of DHT on ER-α-positive breast cancer MCF-7 cells is examined, the androgen stimulates cell proliferation. Treatment with an ERα antagonist, ICI 182,780, and *siRNA* knockdown of ER blocked the proliferative effect of DHT on MCF-7 cells [7]. These results suggest that DHT stimulates MCF-7 cell proliferation via ERα rather than via an AR.

## 4. Integrin αvβ3 as a Receptor for DHT

Although androgen may inhibit the proliferation of breast cancer cells [31,32,33], a stimulatory effect of DHT on the proliferation of triple-negative human breast cancer MDA-MB-231 cells has been observed [7]. Integrin monomer αv antibodies and Arg-Gly-Asp (RGD) peptides inhibit the action of DHT in MDA-MB-231 cells, but are ineffective in MCF-7 cells [7]. Thus, the mechanisms of DHT action differ in ER-positive and -negative breast cancer cell lines, and only in the ER-negative cell lines is there evidence for the existence of a DHT receptor on integrin αvβ3. Studied in prostate cancer and breast cancer cells, ligand-binding to integrin αvβ3 activates FAK, and consequently, FAK, PI-3K, and the Rac1 pathway, leading to the reorganization of actin [34].

Increased FAK activity in tumors has been shown to contribute to phosphorylation of Shc and likely to the promotion of Ras activity, extracellular signal-regulated kinase 2 (ERK2) activation, and cell proliferation in vitro and in vivo [35]. Evidence also indicates that recruitment of an isoform of Shc adaptor proteins, p66Shc, is linked to integrin αvβ3 clustering [35,36,37]. The levels of p66Shc are higher in cancer cells than that in the adjacent non-malignant cells in breast, prostate, ovarian, thyroid, and colon carcinoma tissues [38]. Prostate and ovarian cancer cell proliferation appear to require functional steroid receptors and the elevation of p66Shc protein levels [39].

On the other hand, DHT binds to integrin αvβ3 and stimulates ERα-negative breast cancer proliferation, in which phosphorylation of integrin αvβ3-associated p66Shc is either stimulated by DHT directly or indirectly via the vascular endothelial growth factor (VEGF) signal pathway. In these steroid-treated cells, the level of p66Shc protein is elevated, at least in part due to the inhibition of its ubiquitination [39]. This suggests the existence of a possible therapeutic pathway via the upregulation of ubiquitination of p66Shc protein in advanced cancers.

## 5. Androgens and Breast Cancer Cell Proliferation

Whether androgens are able to induce breast cancer cell proliferation has been a matter of debate. The aromatase activity of breast cancer cells may be sufficient to convert androgen to estrogen and generate local estrogen responses [40]. This process may require the complexation of aromatase and cytochrome P450. This testosterone-induced response of the expression of estrogen-responsive gene pS2 is inhibited by the aromatase inhibitor 7α (4′-amino) phenylthio-1,4-androstadiene-3,17-dione (7α-APTADD) and by 10 µM tamoxifen in breast cancer MCF-7 cells [41]. In the patient on tamoxifen or an aromatase inhibitor who has a recurrent ER-α-positive tumor, it is possible that residual circulating androgen is contributing to breast cancer cell proliferation [42]. To address this issue, the androgen analog specificity of the DHT receptor needs to be determined.

In addition to aromatase pathway, the sulfatase pathway converts estrone sulfate (E1S) into estrone (E1) and into final product E_2_, synthesized by the 17β-hydroxysteroid dehydrogenase type 1 (17β-HSD1). The molecular mechanisms of 17β-HSD1-induced breast cancer growth include estradiol synthesis and DHT inactivation. In addition, 17β-HSD1 can enhance the E_2_-induced expression of endogenous pS2; this suggests involvement of 17β-HSD1 in estrogen responsiveness and breast cancer growth [43].

However, DHT-induced cell proliferation in ER-positive MCF-7 breast cancer cells is inhibited by an ER-α antagonist, ICI 182,780, but not by the AR inhibitor flutamide [7]. DHT may interact with ERs to induce proliferation in ER-α positive breast cancer cells.

## 6. Integrin αvβ3 as a Receptor for Resveratrol

Resveratrol is a comprehensively studied, naturally occurring polyphenol with desirable properties in several biologic models. These activities include cardiovascular protection [44] and remarkable anti-cancer properties [45]. Whether resveratrol can have substantive clinical anticancer properties has repeatedly been subjected to question, because of the agent’s short half-life in the circulation of the intact organism and its rapid intracellular metabolism/turnover rate [46].

### 6.1. Resveratrol-Induced Apoptosis Signal Transduction Pathways: ERK1/2 and AMPK

A cell surface receptor for resveratrol on integrin αvβ3 has been identified by our group [47]. The existence of such a receptor suggests its ability to transduce the plasma and phosphorylated p53-dependent apoptosis. The signal transduction pathway involved in AMP-activated protein kinase (AMPK) activation was subsequently discovered to be associated with the action of resveratrol [48]. It is also remarkable that the receptors for steroid hormones and for resveratrol on the integrin do not appear to interact with one another. Both steroid hormones and resveratrol activate intracellular pools of extracellular signal-regulated kinases 1/2 (ERK1/2), but resveratrol is pro-apoptotic [49] and steroid hormones are anti-apoptotic by ERK1/2-dependent pathways [50]. The pro-apoptotic activity of resveratrol is blocked by Arg-Gly-Asp (RGD) peptides, which bind to the head of the extracellular domain of integrin αvβ3. This suggests that an important binding site for resveratrol may be proximal to the RGD peptide receptor on this integrin. However, recent evidence suggests that the cysteine-rich domain of the integrin may include the binding site for resveratrol.

The role of activated AMPK in cancer cell proliferation is controversial [51,52]. AMPK kinase (AMPKK) is responsive to activate AMPK by phosphorylation at Thr-172. The liver kinase B1 (LKB1) is a serine–threonine kinase that contributes to the regulation of cell energy metabolism, cell proliferation, and cell polarity [53,54]. Cytochrome P450-1A1 (CYP1A1) promotes breast cancer proliferation and survival through the suppression of AMPK signaling [52]. Compound C, an inhibitor of AMPK, promotes apoptotic cell death in various cancer cells; example cells include breast cancer cells and glioma [51]. A pharmacologic analogue of AMP—5-amino-1-β-Dffff-ribofuranosyl-imidazole-4-carboxamide (AICAR)—is an AMPK inhibitor with anticancer properties based upon activation of LKB1 [55]. However, it is not clear that activated AMPK is linked to resveratrol-induced apoptosis.

### 6.2. Resveratrol-Induced Nuclear COX-2

Resveratrol is able to induce the nuclear accumulation of cyclooxygenase-2 (COX-2) [45,49,56,57,58]. An index of tumor cell aggressiveness is build-up of cytoplasmic COX-2 [59,60], whose principal product is prostaglandins. COX-2 inhibition may improve clinical outcomes of certain cancers or be a cancer preventive, in the case of colon carcinoma [61].

Inducible accumulation of nuclear COX-2 is a wholly different biologic product. It is pro-apoptotic, and can interact with Ser-15-phosphorylated p53 and act as a co-activator [47,49,56,57,58]. ERK1/2 activation fosters the nuclear complexation of p53, p300, and COX-2 [49,56]. P300 is a co-activator of pro-apoptotic p53 [47,62], and also supports the accumulation of nuclear hormone receptors [63].

That the pro-apoptotic activity of resveratrol depends upon the nuclear accumulation of COX-2 suggests that pharmacologic COX-2 inhibitors, such as anti-inflammatory agents, also render them as inactivators of the anticancer (pro-apoptotic) activity of resveratrol and other polyphenols. NS-398 is an example of another experimental anti-inflammatory agent that also blocks the resveratrol-induced nuclear accumulation of COX-2 [49,56,57,58,64]. Concomitant administration of such agents with resveratrol may reduce pro-apoptotic activity of the stilbene in clinical applications.

### 6.3. Other Mechanisms Involved in Resveratrol-Induced Anti-Proliferation in Breast Cancers

Induced by resveratrol, apoptosis promoted in MCF-7 breast cancer cells by resveratrol depends upon the downregulation of anti-apoptotic Bcl-2. The mechanism of this downregulation is not clear, but may be linked to mitochondrial membrane actions of the stilbene that increase reactive oxygen species and nitric oxide production [65].

Nuclear factor κB (NF-κB), a regulator of Bcl-2 expression, and calpain protease activity, a regulator of NF-κB, are both inhibited by resveratrol [66,67]. NF-κB and calpain activities are PI-3K-dependent. NF-κB inhibition may result in diminished matrix metalloproteinase (MMP)-9 activity and decreased cell migration. Such observations suggest that resveratrol-induced apoptosis in MCF-7 cells could involve an oxidative, caspase-independent mechanism. The inhibitory effect of resveratrol is mediated in part through the suppression of activation of the PI-3K/Akt signaling pathway, whereby inhibition of PI-3K signaling converges with Bcl-2 through NF-κB and calpain protease activity [66]. Resveratrol also modulates the cell cycle and induces apoptosis in MCF-7 breast tumor cells by interfering with the ERα-dependent PI-3K pathway.

## 7. Interaction of Resveratrol and DHT

Both resveratrol and DHT activate ERK1/2. On the other hand, in breast cancer resveratrol inhibits PI-3K/AKT activation, which is involved in anti-apoptotic pathways and is negatively regulated by phosphatase and tensin homolog (PTEN). In addition, nuclear PTEN affects the cell cycle by negatively regulating the ERK pathway and cyclin D [68,69]. Interestingly, reduced PTEN protein levels are reported in sporadic breast cancers [29,70]. ER-α downregulates the accumulation of PTEN through PI-3K activation in breast cancer cells [71]. The level of PTEN protein in MCF-7 cells is significantly lower than that in MDA-MB 231 cells, and this is correlated with ER-α-positive status in MCF-7 cells. Resveratrol stimulates PTEN expression through AR inhibition [68]. Since DHT via ER-α stimulates ER-α-positive breast cancer proliferation, it is not surprising that DHT decreases PTEN expression and resveratrol increases PTEN expression in breast cancer cell lines, and thus inhibits proliferation [72].

In ER-negative human breast cancer cells, we have already demonstrated the susceptibility to DHT stimulation [7]. Resveratrol inhibits DHT-induced cell proliferation in MDA-MB cells. However, the inhibitory effect of resveratrol on DHT is not at the level of the receptor on integrin αvβ3, since ERK1/2 activation had an additive effect in the combination treatment of resveratrol and DHT. In both androgen-dependent and -independent prostate cancer cells, resveratrol inhibits AR transcriptional activity, but does not affect the total and nuclear AR levels [73]. Thus, the inhibitory effects of resveratrol on AR activity result from mechanisms other than AR nuclear translocation [74]. Resveratrol inhibits the binding of AR to the enhancer region of prostate-specific antigen (PSA) and decreases the acetylation of AR [75], although other studies suggest that resveratrol may not affect AR binding to DNA [74]. Resveratrol reduces the production of PSA, a notable target gene of AR. Resveratrol treatment also decreases the mRNA level of AR-regulated genes, such as *NKX 3.1*.

The interaction between steroid hormones and growth factors plays an important role in breast cancer development. DHT increases both epidermal growth factor receptor (EGFR) numbers and receptor–ligand affinity in androgen-sensitive prostate cancer cells; this correlates with increased EGF binding and an enhanced mitogenic response to EGF [76,77]. DHT up-regulates the levels of phosphorylation of EGFR (pEGFR) and its downstream proteins AKT (pAKT) and ERK1/2 (pERK) in AR-positive cells. However, the expression of EGFR in human breast cancer tissues has an inverse relationship with expression of the ER-α, and may be associated with a poor clinical outcome [78]. Thus, cross-talk between EGF and DHT may be more dominant in ER-negative than in ER-positive breast cancer cells. In addition to inhibiting DHT-induced signal transduction and biological activities, resveratrol is able to directly bind to EGFR and inhibit EGFR phosphorylation [74]. A summary of resveratrol- and DHT-affected signal transduction is listed in Table 2.

## 8. Effect of DHT and Resveratrol on Metastasis

Metastasis is the primary cause of death in breast cancer patients. Cell migration and invasion play important roles in neoplastic metastasis. Cell proliferation, differentiation, apoptosis, and cell motility are prompted and controlled by a host of growth factors and hormones. The extracellular matrix (ECM) of human breast tumor cells has several effects of such cells: ECM is mitogenic for fibroblasts, and also stimulates the synthesis of collagen and elastin [79,80]. Both effects contribute to the desmoplastic response to human breast cancer in situ [79,80]. Maintenance of the differentiated state, including hormone and growth factor responsiveness, requires extracellular matrix proteins as a substrate for cells. The metastatic spread of cancer cells involves a complex process of detachment via anti-adhesion molecules and attachment and migration through adhesion [81]. In addition, DHT modulates the mechanoreception of human osteoblastic cells. DHT modulates the expression of adhesion molecules, such as fibronectin and the fibronectin receptor [82]. Some effects of DHT and resveratrol on receptors that play a role in metastasis are listed in Table 3.

## 9. Integrin αvβ3 and Metastasis

Integrin αvβ3 in the endothelial cell membrane is essential to the migration of capillaries into cancer tissue. This integrin is also a survival factor for endothelial cells [83]. The expression of integrin αvβ3 appears to play a key role in the development of bone metastasis from breast cancer [84]. Treatment with DHT downregulates the cell surface expression of integrin α2β1, but has little effect on the levels of integrin α3β1 and α5β1 in prostate cancer PC-3 cells containing transfected ARs [81]. Androgen also decreases the adhesion of AR-transfected PC-3 cells to collagen type I. Integrins αvβ3 and αvβ5 are critical components of the process of angiogenesis, and are a rationale for multiple attempts to base therapeutic anti-angiogenesis on integrin antagonists [85]. Downstream transduction of signals generated at surface αvβ3 importantly regulates VEGF expression in breast cancer [85]. Integrin αvβ3 clustering promotes the recruitment of p66Shc, and subsequently the phosphorylation of β3-associated p66Shc to upregulate VEGF expression. An important facet of mediation by integrin αvβ3 of VEGF expression and cancer-related angiogenesis is the phosphorylation of p66Shc [44]. In urinary bladder cancer patients, castration reduces tumor cell growth by DHT in vivo and decreases thrombospondin-1 (TSP1) expression [86]. Resveratrol has been shown to interact with the integrin β3 subunit, raising the possibility that inhibition of endothelial αvβ3 integrin function may contribute to the stilbene’s angiosuppressive activity [87]. Via ER-α, resveratrol increases the interaction between caveolin-1 (Cav-1) and c-Src, and increases the phosphorylation of Cav-1, c-Src, and eNOS in human umbilical vein ECs (HUVECs) [88]. In vivo, the angiogenesis of chick embryo area vasculosa and of mouse B16 melanoma are subject to inhibition by resveratrol. The polyphenol also blocks integrin-dependent vascularization models, such as αvβ3-linked endothelial wall adhesion and migration of integrin monomer β3 in focal adhesion contacts [87]. The latter may be relevant to management of ER-negative breast cancer.

## 10. VEGF and Metastasis

Integrin αvβ3-associated signaling regulates the growth of both prostate and breast tumors by influencing vascular endothelial growth factor (VEGF) expression [85]. Androgenic regulation of VEGF gene expression occurs shortly after androgen stimulation [89]. DHT importantly upregulates VEGF mRNA abundance [90], and VEGF biological activity is increased by DHT. Androgen regulates prostate blood flow, and VEGF is involved in blood flow regulation, with an activity equal to that of DHT.

Levels of the p66Shc protein are increased in cell lines with highly metastatic ability and in lymph node-positive tumors [91]. Downregulation of p66Shc inhibits VEGF expression, as well as tumor growth and angiogenesis in vivo [36]. Androgens have indirect effects on these cells via the upregulation of stromal VEGF production and angiogenesis. The use of VEGF inhibition as a substitute for anti-androgenic therapy may be effective against prostate diseases, especially disease that is relatively independent of androgens and that is hypervascular. VEGF-induced new blood vessel formation that is a function of reactive oxygen species (ROS)-dependent non-receptor protein tyrosine kinase (SRC kinase) activation is also inhibited by resveratrol [92].

## 11. Conclusions

The regulation of breast cancer cell proliferation is conventionally regarded as a function of the degree to which ER-positive cells have access to estrogen and to systemic polypeptide growth factors. Beyond surgery, tumor irradiation, and chemotherapy, the management of breast cancer emphasizes the long-term suppression of the action of endogenous estrogen with tamoxifen, or inhibition of estrogen synthesis with aromatase inhibition. Non-genomically, estrogen may support breast tumor growth through ER-like proteins in the membrane. This is now under investigation [93].

**Figure 1:**
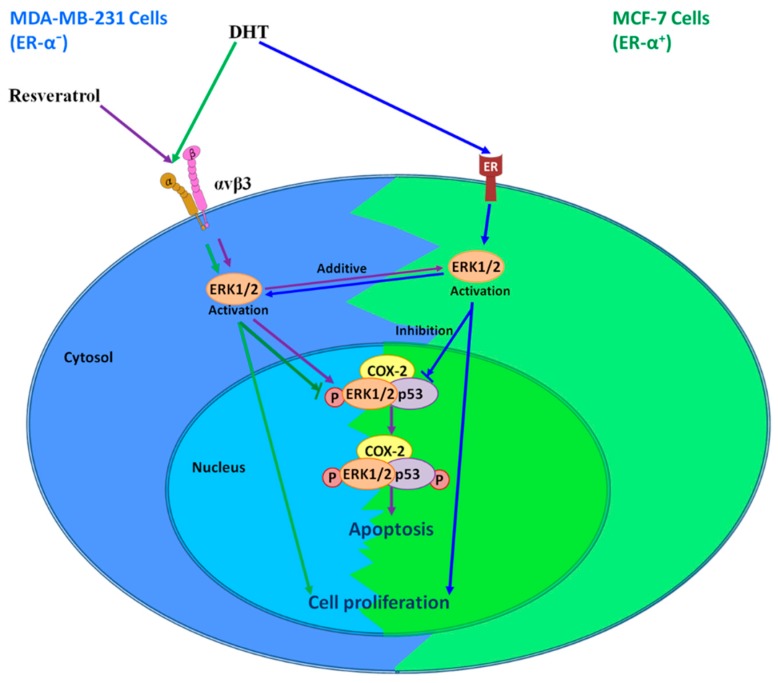
Signal transduction pathways in the actions of resveratrol and DHT in different breast cancer cells. Extracellular signal-regulated kinases 1/2 (ERK1/2) activated at the plasma membrane are a result of resveratrol binding, and predictably result in cyclooxygenase-2 (COX-2) expression. Newly-generated COX-2 complexes with phosphorylated ERK1/2 (pERK1/2), is subject to SUMOylation, and then translocates to the nuclear compartment. In the nucleus, COX-2 and modified/activated p53 act as a transcription factor complex, causing the expression of p53-responsive genes. On the other hand, DHT binds to membrane estrogen receptor (ER)-α in ER-α-positive breast cancer cells, while it binds to integrin αvβ3 in ER-α-negative breast cancer cells. DHT activates ERK1/2 and induces cell proliferation. Activated ERK1/2 has discrete functions, depending upon whether activation in cancer cells is a response to resveratrol or DHT. ERK1/2 activation in response to resveratrol causes apoptosis. In contrast, DHT-activated ERK1/2 disrupts resveratrol-induced anti-proliferation. P: phosphorylation. ↓: active, ↓: inhibit.

Identification of the resveratrol receptor site on integrin oteinon breast cancer cells and other solid tumor cells provides useful insights into the actions of this stilbene. Resveratrol binds to the integrin αvβ3 and rapidly activates ERK1/2 and AMPK to initiate the nuclear accumulation of COX-2 and p53-dependent apoptosis (Figure 1), regardless of uptake of the compound and chemical processing, which have been widely studied.

DHT actins via integrin αvβ3 (Figure 1), behaving as a trophic agent for certain types of breast cancer cells [8]. Remarkably, the mechanisms involved in DHT-induced proliferative action differ between ER-α-negative and ER-α-positive cells in vitro. Receptors for DHT on integrin αvβ3 on the cell surface are required for the proliferative effect of the androgen in ER-negative cell; however, this may be irrelevant to ER-positive cells, whose cell surface ERs are required for the action of DHT. On the other hand, nuclear ARs may not play a role in the mechanism of DHT action in either type of cell [94]. What is somewhat surprising is that resveratrol-induced anti-proliferation is blocked by DHT, via a discrete receptor on the integrin. It is possible that the separate receptors for DHT and for resveratrol on the integrin can be modulated/inhibited to permit unimpeded expression of the anticancer actions of resveratrol at integrin αvβ3. It will also be useful to determine whether the resveratrol receptor on the integrin is related to the stilbene’s enhancement of breast cancer cell retention of doxorubicin [95], and whether this receptor mediates downregulation of DNA repair genes in tumor cells by resveratrol [96]. This is relevant to the radiosensitivity/radio-resistance of cancer cells. Currently, sulforaphane (SFN), epigallocatechin-3-gallate (EGCG), and other herb medicines have been shown to increase ER-α expression in ER-negative breast cancer MDA-MB cells [97,98]. However, resveratrol’s potential has not been examined yet. To understand the role of integrin αvβ3 in DHT and resveratrol-induced biologic activities in breast cancer should help with the clinical manipulation of breast cancers.

## Figures and Tables

**Table 1:** Hormone receptors and their functions in cancer cells [7,8,9,10,11,12,13].

Hormone	Receptor	Functions	References
Estrogen	Estrogen Receptor-α (ER-α)	To form ligand-ER complex and controlling gene expression. To stimulate proliferation of breast cancer cells	[7]
	Integrin αvβ3	NA	[8]
DHT	Androgen Receptor (AR)	To form ligand-AR complex and controlling gene expression To stimulate proliferation of prostate cancer cells.	[9]
	Estrogen Receptor-α (ER-α)	To stimulate proliferation of ER-positive breast cancer cells	[7]
	Integrin αvβ3	To stimulate proliferation of ER-negative breast cancer cells	[10]
Thyroid hormone	Thyroid hormone Receptor-α (TR-α)	To stimulate cancer cell growth	[11]
	Thyroid hormone Receptor-β (TR-β)	To inhibit cancer cell growth, however, mutant TR-β may activate cancer cell growth	[12]
	Integrin αvβ3	To stimulate cancer cell growth	[13]

**Table 2:** Effect of resveratrol and dihydrotestosterone (DHT) on the signal transduction pathway in cancer cells.

	Resveratrol	DHT
Binding Site	Integrin αvβ3	Integrin αvβ3/ER-α/AR
ERK1/2	↑	↑
PI-3K	↓	↑
AKT	↓	↑
PTEN	↑	↓

↑: increase, ↓: decrease.

**Table 3:** Effects of resveratrol and DHT on the expression of receptors and activities in cancer cells.

	Resveratrol	DHT
Integrin	β3 ↑	α2β1 ↓
EGFR	↓	↑
VEGFR	─	↑
VEGF	↓	↑

↑: increase, ↓: decrease, ─: no effect.

## Abbreviations

ACTN4	Actin-binding protein, actinin α 4
AMPK	AMP-activated protein kinase
AMPKK	AMP-activated protein kinase kinase
7α-APTADD	7α (4′-amino) phenylthio-1,4-androstadiene-3,17-dione
AR	Androgen receptor
BRCA1	Breast cancer 1 gene
Cav-1	Caveolin-1
COX-2	Cyclooxygenase-2
CYP1A1	Cytochrome P450-1A1
DHEAS	Dehydroepiandrosterone sulfate
DHT	Dihydrotestosterone
E1	Estrone
E1S	Estrone sulfate
EGFR	Epidermal growth factor receptor
ER	Estrogen receptor
ERK1/2	Extracellular signal-regulated kinases
FAK	Focal adhesion kinase
HER2	Human epidermal growth factor receptor 2
17β-HSD1	17β-hydroxysteroid hydrogenase type 1
iARs	Classical intracellular androgen receptors
LKB1	Liver kinase B1
MAPK	Mitogen-activated protein kinase
mARs	Membrane androgen receptors
MMP-2	Matrix metalloproteinase-2
PI-3K	Phosphatidylinositol-3-kinase
PKC δ	Protein kinase C δ
PR	Progesterone receptor
PTEN	Phosphatase and tensin homolog
RGD	Arg-Gly-Asp
TSP1	Thrombospondin-1
VEGF	Vascular endothelial growth factor
